# Highly Pathogenic Avian Influenza A(H5N6) Virus Clade 2.3.4.4h in Wild Birds and Live Poultry Markets, Bangladesh

**DOI:** 10.3201/eid2709.210819

**Published:** 2021-09

**Authors:** Jasmine C.M. Turner, Subrata Barman, Mohammed M. Feeroz, M. Kamrul Hasan, Sharmin Akhtar, Trushar Jeevan, David Walker, John Franks, Patrick Seiler, Nabanita Mukherjee, Lisa Kercher, Pamela McKenzie, Tommy Lam, Rabeh El-Shesheny, Richard J. Webby

**Affiliations:** St. Jude Children’s Research Hospital, Memphis, Tennessee, USA (J.C.M. Turner, S. Barman, T. Jeevan, D. Walker, J. Franks, P. Seiler, N. Mukherjee, L. Kercher, P. McKenzie, R. El-Shesheny, R.J. Webby);; Jahangirnagar University, Savar, Bangladesh (M.M. Feeroz, M.K. Hasan, S. Akhtar);; The University of Hong Kong School of Public Health, Hong Kong, China (T. Lam);; National Research Centre, Giza, Egypt (R. El-Shesheny)

**Keywords:** highly pathogenic avian influenza, HPAI, H5N6, influenza, Bangladesh, live poultry markets, wild birds, poultry, viruses, respiratory infections

## Abstract

Migratory birds play a major role in spreading influenza viruses over long distances. We report highly pathogenic avian influenza A(H5N6) viruses in migratory and resident ducks in Bangladesh. The viruses were genetically similar to viruses detected in wild birds in China and Mongolia, suggesting migration-associated dissemination of these zoonotic pathogens.

Highly pathogenic avian influenza (HPAI) A(H5) viruses were identified in 1996 in a goose from Guangdong, China, and the evolution of the hemagglutinins (HAs) of these A/goose/Guangdong/1/96 (Gs/GD) lineage viruses has given rise to multiple genetically distinct phylogenetic clades (*1*). The emergence of HA clade 2.3.4.4 viruses was associated with several different virus subtypes, including H5N6 (*2*). As of March 2021, a total of 29 laboratory-confirmed human cases of H5N6 viruses have been reported from China, and 9 patients have died (*3*). Clade 2.3.4.4 H5N6 viruses have subsequently evolved, requiring further clade designations. Clade 2.3.4.4h viruses are found in China, Laos, and Vietnam (*4*). In December 2019 and January 2020, 2.3.4.4 H5N6 viruses were isolated from dead migratory whooper swans (*Cygnus cygnus*) and mute swans (*Cygnus olor*) in Xinjiang, western China (*5*). In April 2021, the same virus was detected in migratory birds in Mongolia (*6*).

In Bangladesh, HPAI A(H5) viruses have been in circulation since 2008; the predominant clades found are 2.2.2 and 2.3.2.1a. HPAI A(H5N6) clade 2.3.4.4b viruses were identified in domestic poultry in Bangladesh in 2016 (*7*,*8*). Although the viruses were detected in live poultry markets (LPMs), they did not replace the H5N1 viruses in circulation, and as of April 2021, there have been no more reports of H5N6 virus detection (*9*,*10*). We report a new introduction of clade 2.3.4.4.h viruses that are similar to viruses detected in China (Xinjiang) and Mongolia (*5*,*6*), suggesting that migratory birds of the Central Asian flyway introduced this virus into Bangladesh.

## The Study

Since 2015, our active surveillance in Bangladesh has been ongoing in both LPMs and Tanguar Haor, a wetlands area where local domestic ducks are reared and where birds winter during the migratory season (Appendix, Table 1). We collected H5N6 virus–positive oropharyngeal and cloacal swabs from 2 apparently healthy wild birds in Baghmara, Tanguar Haor: a ferruginous duck on January 19, 2020, and a common pochard on January 20, 2020. We also obtained positive fecal samples from wild mallard ducks on January 26, 2020, in Puran Gao, Tanguar Haor. The next day, we obtained positive oropharyngeal and cloacal swabs from apparently healthy Khaki Campbell ducks located on various farms in Golabari, Tanguar Haor (Appendix Table 1). On February 18, 2020, ≈3 weeks after detection of H5N6 virus in Tanguar Haor, an apparently healthy mallard duck located in a Dhaka LPM was also found to be infected with H5N6. Surveillance conducted on February 22, 2020, on various farms in Chitergao, Tanguar Haor, revealed an additional 24 more apparently healthy Khaki Campbell ducks infected with H5N6 virus. During our surveillance study, we identified a total of 40 domestic and wild birds infected with H5N6 virus clade 2.3.4.4h during January–February 2020 (Appendix Table 1).

We determined the complete genome sequences of the 40 HPAI A(H5N6) viruses. The sequence similarity between viruses was 99.4%–100%. As a representative virus, A/Ferruginous duck/Bangladesh/42380/2020 (H5N6) had a high nucleotide identity (99.6%–99.9%) to the HPAI A(H5N6) viruses of clade 2.3.4.4h from China (Xinjiang, January 2020) and Mongolia (April 2020) (Table).

**Table:** Nucleotide sequence identities between the A/Ferruginous duck/Bangladesh/42380/2020 (H5N6) virus from Bangladesh and nearest virus homologs*

Gene	GenBank accession no.	Virus	% Identity
PB2	MT872369.1	A/Whooper swan/Mongolia/25/2020 (H5N6)	99.83
	MW108029.1†	A/duck/Hunan/1.12_YYGK74H3-OC/2018 (H5N6)	98.65
PB1	MT872369.1	A/Whooper swan/Mongolia/25/2020 (H5N6)	99.87
	MW104086.1	A/chicken/Guangdong/7.20_DGCP022-O/2017 (H5N6)	99.04
PA	EPI_ISL_418181	A/Whooper swan/Xinjiang/13/2020 (A/H5N6)	99.9
	EPI_ISL_340825	A/Env/Guangdong/Jieyang/C18289059/2018(H5N6)	99.5
HA	EPI_ISL_418175	A/Whooper swan/Xinjiang/7/2020 (A/H5N6)	99.8
	EPI_ISL_340844	A/Env/Guangdong/C17285752/QY/2017 (H5N6)	98.9
NP	MT872369.1	A/Whooper swan/Mongolia/25/2020 (A/H5N6)	99.65
	MW108029.1	A/duck/Hunan/1.12_YYGK74H3-OC/2018 (H5N6)	99.64
NA	EPI_ISL_418181	A/Whooper swan/Xinjiang/13/2020 (A/H5N6)	99.9
	MW108138.1	A/duck/Hunan/11.30_YYGK63E3-OC/2017 (H5N6)	99.36
M	MT872369.1	A/Whooper swan/Mongolia/25/2020 (H5N6)	99.6
	EPI_ISL_340825	A/Env/Guangdong/Jieyang/C18289059/2018 (H5N6)	99.9
NS	EPI_ISL_418181	A/Whooper swan/Xinjiang/13/2020 (A/H5N6)	99.9
	MW108029.1	A/duck/Hunan/1.12_YYGK74H3-OC/2018 (H5N6)	99.29

*HA, hemagglutinin; MP, matrix protein; NA, neuraminidase; NP, nucleoprotein; NS, nonstructural protein; PA, acidic polymerase; PB1, basic polymerase 1; PB2, basic polymerase 2. †Nearest virus homologs to A/Ferruginous duck/Bangladesh/42380/2020 (H5N6) excluding the H5N6 viruses from China (Xinjiang), and Mongolia.

**Figure 1:**
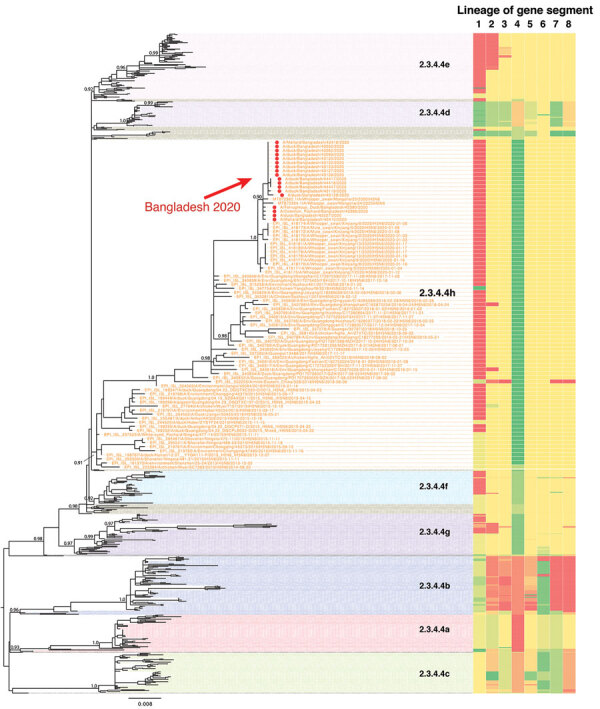
Phylogenetic tree of H5N6 viruses sequenced in this study, in addition to all publicly available H5N6 and closely related H5 sequences available from GenBank and GISAID. Red dots represent the Bangladesh H5N6 viruses sequenced in this study. Topological support values (SH-like support) of selected nodes are displayed.

**Figure 2:**
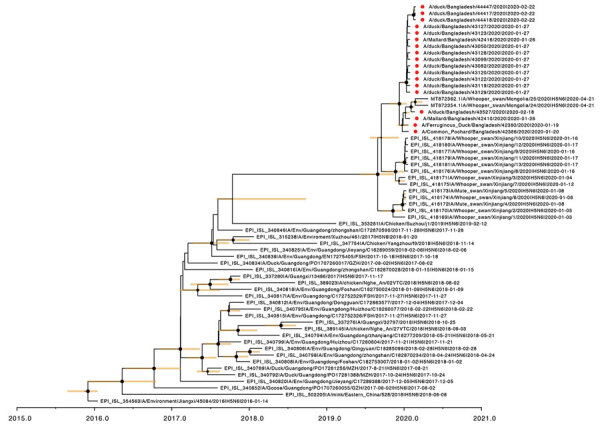
Time to the most recent common ancestor of Bangladesh H5N6 viruses; maximum clade credibility temporal phylogeny of the hemagglutinin (HA) gene. The H5N6 viruses from Bangladesh are represented by red dots. Posterior clade probabilities are indicated by the sizes of the internal node circles. Shaded bars represent the 95% highest probability distribution for the age of each node with posterior clade probability >0.3.

An outbreak of H5N6 virus clade 2.3.4.4h in whooper swans in China (Xinjiang) and Mongolia in early 2020 suggested potential further distribution of these viruses across Asia, especially to areas where poultry is raised along the migration routes of wild birds. We combined genome sequences generated in this study with all sequences of H5N6 viruses available in GenBank and the GISAID database (*11*). Phylogenetic analysis confirmed that the Bangladeshi A(H5N6) isolates are of clade 2.3.4.4h and clustered with the recent HPAIV A(H5N6) isolates from whooper swans in Xinjiang, western China and in Mongolia (Figure 1). The time of most recent common ancestry for HPAI A(H5N6) viruses (Figure 2) suggests that the viruses from China, Mongolia, and Bangladesh share a common ancestor of unknown origin that emerged around mid-2019.

The phylogenetic clustering observed for the H5 gene was also conserved for the remaining 7 genes; the viruses from Bangladesh, China, and Mongolia were of the same genotype, with no evidence of reassortment (Appendix Figure). The A(H5N6) viruses from Bangladesh shared genetic features with their homologs from China, including an HA cleavage site, PLRERRRKR/G, which is characteristic of high pathogenicity in chickens (Appendix Table 2). We also found an amino acid deletion at position 133 in the HA protein (H3 numbering) in all our isolates, a feature common with clade 2.3.4.4.h isolated from humans (Appendix Table 2) and associated with alteration of the H5 HA receptor binding pocket (*12*). Deletions were also present in both neuraminidase (NA) (an 11-aa deletion in the stalk region) and nonstructural protein 1 (NS1) (deletion from residues 80–84; Appendix Table 2), which are associated with high pathogenicity in avian hosts (*13*). Postinfection ferret antisera raised to A/duck/Bangladesh/43127/2020 (H5N6) reacted to the World Health Organization’s candidate clade 2.3.4.4h vaccine virus, A/Guangdong/18SF020/2018 and, as expected, to all Bangladesh H5N6 viruses tested (Appendix Table 3).

Migratory birds are key in the evolution, maintenance, and spread of avian influenza viruses. We have previously identified viruses in LPMs after their detection in wild birds and domestic ducks in Tanguar Haor (*8*,*14*,*15*). Similarly, detection of the H5N6 virus in an LPM after detection in Tanguar Haor highlights the continuum of migratory birds of the Central Asian flyway and domestic ducks in Tanguar Haor as vectors for viral movement at the wild bird–poultry interface. We also detected a duck that was co-infected with A/duck/Bangladesh/44500/2020 (H10N7) and A/duck/Bangladesh/44500/2020 (H5N6), raising the possibility of reassortment and highlighting the potential effect of this genetic diversification.

## Conclusions

We have identified HPAIV A(H5N6) viruses from migratory birds, domestic duck farms, and LPMs in Bangladesh at a similar time to their detection in China and Mongolia. The wider distribution of this group of viruses with documented zoonotic potential is cause for considerable public health concern. Monitoring for their establishment in South Central Asia must be intensified.

Appendix Additional information on highly pathogenic avian influenza A(H5N6) in wild birds and poultry markets, Bangladesh.
