# Supplementary material for: Highly Pathogenic Avian Influenza A(H5N6) Virus Clade 2.3.4.4h in Wild Birds and Live Poultry Markets, Bangladesh
Source: Emerg Infect Dis. 2021 Sep;27(9):2492–4. doi: 10.3201/eid2709.210819 (PMC8386775; doi:10.3201/eid2709.210819)
Supplement: Appendix — Additional information on highly pathogenic avian influenza A(H5N6) in wild birds and poultry markets, Bangladesh. [file 21-0819-Techapp-s1.pdf]

Article DOI: https://doi.org/10.3201/eid2709.210819

Highly Pathogenic Avian Influenza A(H5N6) Virus Clade 2.3.4.4h in Wild Birds and Live Poultry Markets, Bangladesh

Appendix

Methods

Isolation of A(H5N6) from Live Poultry Markets and Wild Birds, Bangladesh

Sample Collection

During the wild bird migratory season (December 2019–March 2020), we collected 1,000 samples monthly from free-range farm ducks (500) and wild birds (500) in Tanguar Haor, Bangladesh. In December 2019 and February 2020, we collected 160 poultry samples in live poultry markets (LPMs) in or near Dhaka. Samples collected from LPMs in or near Dhaka, (oropharyngeal, cloacal, and water samples) were obtained primarily from ducks, chickens, and quail. We also collected domestic duck samples from various farms in Tanguar Haor. In addition to the duck samples, we collected fecal samples and, on several occasions, oropharyngeal and cloacal samples from various species of wild birds within the Tanguar Haor area. Samples were stored at ~4°C in the field and moved to liquid nitrogen within 1 week of collection. Samples were shipped on a routine basis to the biosafety level-3 facilities of St. Jude Children's Research Hospital (Memphis, TN, USA).

Sample Screening and Virus Isolation

We screened all samples for influenza by rRT-PCR for the presence of the matrix gene . All matrix gene positive samples from LPMs were subsequently screened for H5; all matrix gene positive samples from Tanguar Haor were inoculated in 10-day old embryonated chicken eggs for isolation. We chosen samples from LPMs for virus isolation on the basis of selection criteria that included date of collection, host species, market location, and rRT-PCR cycle threshold (Ct)

values for both matrix gene and/or H5. Swabs from which virus isolates were obtained were subtyped, as described previously (*1*,*2*).

Deep Amplicon Sequencing and Genetic Analysis

We extracted viral RNA using an RNeasy kit (QIAGEN, https://www.qiagen.com); we then performed conventional 2-step RT-PCR using a SuperScript IV first-strand synthesis kit (Invitrogen, https://www.thermofisher.com) with the Uni12 influenza primer. Multiplex PCR of all 8 gene segments was conducted by using Phusion high-fidelity DNA polymerase (New England Biolabs, https://www.neb.com) with the Uni12/13 primers. We purified PCR products using Illustra GFX PCR DNA and gel band purification kit (GE Healthcare, https://www.gehealthcare.com). DNA libraries were prepared by the staff of the Hartwell Center at St. Jude Children's Research Hospital by using NEXTera XT DNA-Seq library preparation kits (Illumina, https://www.illumina.com) according to the manufacturer's instructions. Pooled libraries were sequenced with an Illumina MiSeq personal genome sequencer by 150-bp paired-end reads. CLC Genomics Workbench, version 20 (CLC Bio, QIAGEN), was used to analyze and process the sequencing reads. DNA Lasergene 15 and BioEdit7.0 (*3*) were used for multiple sequence alignment and genomic signature analysis using the Clustal W algorithm (*4*). MEGA 7 was used for the phylogenetic tree reconstruction by applying the neighbor-joining method with Kimura's two-parameter distance model and 1,000 bootstrap replicates (*5*).

Antigenic Characterization

We used hemagglutination inhibition (HI) assay to antigenically characterize the viruses. The panel of antisera used in the HI assay included representatives from the currently circulating genetic sublineages of clade 2.3.3.4. The antiserum against A/duck/Bangladesh/43127/2020 (H5N6) was generated for this study. In brief, ferrets were intranasally infected with 1 mL of 10^6 EID50/mL viruses and then boosted after 3 weeks by intramuscular injection of virus with adjuvant. We collected blood 1 week later for serum isolation. The HI test was performed according to World Health Organization protocols (*6*).

References

1. Barman S, Marinova-Petkova A, Hasan MK, Akhtar S, El-Shesheny R, Turner JCM, et al. Role of domestic ducks in the emergence of a new genotype of highly pathogenic H5N1 avian influenza A viruses in Bangladesh. Emerg Microbes Infec. 2017;6:1–13. https://doi.org/10.1038/emi.2017.60

2. Turner JCM, Feeroz MM, Hasan MK, Akhtar S, Walker D, Seiler P, et al. Insight into live bird markets of Bangladesh: an overview of the dynamics of transmission of H5N1 and H9N2 avian influenza viruses. Emerg Microbes Infec. 2017;6:e12. https://doi.org/10.1038/emi.2016.142

3. Hall TA. BioEdit: a user-friendly biological sequence alignment editor and analysis program for Windows 95/98/NT. Nucleic acids symposium series. London: Information Retrieval Ltd.; 1999. p. 95–8.

4. Thompson JD, Higgins DG, Gibson TJ. CLUSTAL W: improving the sensitivity of progressive multiple sequence alignment through sequence weighting, position-specific gap penalties and weight matrix choice. Nucleic Acids Res. 1994;22:4673–80. PubMed https://doi.org/10.1093/nar/22.22.4673

5. Kumar S, Stecher G, Tamura K. MEGA7: Molecular Evolutionary Genetics Analysis Version 7.0 for bigger datasets. Mol Biol Evol. 2016;33:1870–4. PubMed https://doi.org/10.1093/molbev/msw054

6. World Health Organization. WHO manual on animal influenza diagnosis and surveillance. 2002 [cited 2021 Mar 26]. http://whqlibdoc.who.int/hq/2002/WHO_CDS_CSR_NCS_2002.5.pdf

Appendix Table 1. Summary of surveillance of HPAI A(H5N6) viruses isolated from the Tanguar Haor region and live poultry markets, Bangladesh, 2020

Collection period	Sample collection location	No. samples collected	Isolate host (species)	No. isolates obtained	GenBank accession nos.
Jan 18–20	Tangua Haor wetlands*	15	Ferruginous duck (*Aythya nyroca*); common pochard (*Aythya ferina*)	2	MW466111–7, MW466362–8, MW467515, MW467527
Jan 26–27	Tangua Haor wetlands*	485	Mallard duck (*Anas platyrhynchos*)	2	MW465993–9, MW466070–3, MW467526
Jan 27–28	Tangua Haor duck farm†	500	Domestic duck (*Anas sp.*)	11	MW466048–54, MW466090–6, MW466104–10, MW46615218–24, MW466238–44, MW466252–8, MW466274–80, MW466297–303, MW466355–61, MW466392–8, MW467517, MW467518, MW467520–2, MW467529–31, MW467534, and MW467537
Feb 17–19	Live bird market	160	Domestic duck (*Anas sp.*)	1	MW466151–7, MW467514
Feb 20–22	Tangua Haor duck farm†	500	Domestic duck (*Anas sp.*)	24	MW749019–31, MW749033–9, MW749063–123, MW749132–79, MW749188–94, MW749718–24, MW749733–9, MW749748, MW749751, MW749756, MW749757, MW749759, MW749782–7, MW751428, MW751429, MW751433–5, MW751437, MW751440, MW751442–4, MW751447, MW751448, MW751450–5
Total		1,660		40	

*14 different locations in the wetlands region where fecal samples from migratory birds were collected.
†19 different farms where oropharyngeal and cloacal samples were collected from domestic ducks.

Appendix Table 2. Comparison of amino acid sequences of H10 AIVs isolated from wild birds and live poultry markets, Bangladesh

	PB2			PB1-F2	HA							NA				M2	NS			
						HA del.	Receptor binding								Stalk		NS del.			PDZ
Viruses	E627K	D701N	K482R	Expression	Cleavage site	133*	E190D	N193K	Q226L	G228S	S227N/R	E119D†	H274Y	R292K	del.	S31N	80–84	P42S	D92E	motif
A/Ferruginous duck/Bangladesh/42380/2020	E	D	K	No	RERRRKR↓G	Yes	E	S	Q	G	R	E	H	R	Yes	S	Yes	S	E	ESEV
A/Common pochard/Bangladesh/42386/2020	E	D	R	No	RERRRKR↓G	Yes	E	S	Q	G	R	E	H	R	Yes	S	Yes	S	E	ESEV
A/environment/Bangladesh/42410/2020	E	D	K	No	RERRRKR↓G	Yes	E	S	Q	G	R	E	H	R	Yes	S	Yes	S	E	ESEV
A/environment/Bangladesh/42416/2020	NA	NA	NA	NA	RERRRKR↓G	Yes	E	S	Q	G	R	E	H	R	Yes	S	Yes	NA	NA	NA
A/duck/Bangladesh/43050/2020	E	D	K	No	RERRRKR↓G	Yes	E	S	Q	G	R	E	H	R	Yes	S	Yes	S	E	ESEV
A/duck/Bangladesh/43082/2020	E	D	K	No	RERRRKR↓G	Yes	E	S	Q	G	R	E	H	R	Yes	S	Yes	S	E	ESEV
A/duck/Bangladesh/43099/2020	E	D	K	No	RERRRKR↓G	Yes	E	S	Q	G	R	E	H	R	Yes	S	Yes	S	E	ESEV
A/duck/Bangladesh/43119/2020	E	D	K	No	RERRRKR↓G	Yes	E	S	Q	G	R	E	H	R	Yes	S	Yes	S	E	ESEV
A/duck/Bangladesh/43120/2020	E	D	K	No	RERRRKR↓G	Yes	E	S	Q	G	R	E	H	R	Yes	S	Yes	S	E	ESEV
A/duck/Bangladesh/43122/2020	E	D	K	No	RERRRKR↓G	Yes	E	S	Q	G	R	E	H	R	Yes	S	Yes	S	E	ESEV
A/duck/Bangladesh/43123/2020	E	D	K	No	RERRRKR↓G	Yes	E	S	Q	G	R	E	H	R	Yes	S	Yes	S	E	ESEV
A/duck/Bangladesh/43127/2020	E	D	K	No	RERRRKR↓G	Yes	E	S	Q	G	R	E	H	R	Yes	S	Yes	S	E	ESEV
A/duck/Bangladesh/43128/2020	E	D	K	No	RERRRKR↓G	Yes	E	S	Q	G	R	E	H	R	Yes	S	Yes	S	E	ESEV
A/duck/Bangladesh/43129/2020	E	D	K	No	RERRRKR↓G	Yes	E	S	Q	G	R	E	H	R	Yes	S	Yes	S	E	ESEV
A/duck/Bangladesh/43527/2020	E	D	K	No	RERRRKR↓G	Yes	E	S	Q	G	R	E	H	R	Yes	S	Yes	S	E	ESEV
A/duck/Bangladesh/44417/2020	E	D	K	No	RERRRKR↓G	Yes	E	S	Q	G	R	E	H	R	Yes	S	Yes	S	E	ESEV
A/duck/Bangladesh/44418/2020	E	D	K	No	RERRRKR↓G	Yes	E	S	Q	G	R	E	H	R	Yes	S	Yes	S	E	ESEV
A/duck/Bangladesh/44423/2020	E	D	K	No	RERRRKR↓G	Yes	E	S	Q	G	R	E	H	R	Yes	S	Yes	S	E	ESEV
A/duck/Bangladesh/44424/2020	E	D	K	No	RERRRKR↓G	Yes	E	S	Q	G	R	E	H	R	Yes	S	Yes	S	E	ESEV
A/duck/Bangladesh/44430/2020	E	D	K	No	RERRRKR↓G	Yes	E	S	Q	G	R	E	H	R	Yes	S	Yes	S	E	ESEV
A/duck/Bangladesh/44432/2020	E	D	K	No	RERRRKR↓G	Yes	E	S	Q	G	R	E	H	R	Yes	S	Yes	NA	NA	NA
A/duck/Bangladesh/44433/2020	E	D	K	No	RERRRKR↓G	Yes	E	S	Q	G	R	E	H	R	Yes	S	Yes	S	E	ESEV
A/duck/Bangladesh/44434/2020	E	D	K	No	RERRRKR↓G	Yes	E	S	Q	G	R	E	H	R	Yes	S	Yes	NA	NA	NA
A/duck/Bangladesh/44440/2020	E	D	K	No	RERRRKR↓G	Yes	E	S	Q	G	R	E	H	R	Yes	S	Yes	S	E	ESEV
A/duck/Bangladesh/44442/2020	E	D	K	No	RERRRKR↓G	Yes	E	S	Q	G	R	E	H	R	Yes	S	Yes	NA	NA	NA
A/duck/Bangladesh/44447/2020	E	D	K	No	RERRRKR↓G	Yes	E	S	Q	G	R	E	H	R	Yes	S	Yes	S	E	ESEV
A/duck/Bangladesh/44448/2020	E	D	K	No	RERRRKR↓G	Yes	E	S	Q	G	R	E	H	R	Yes	S	Yes	S	E	ESEV
A/duck/Bangladesh/44453/2020	E	D	K	No	RERRRKR↓G	Yes	E	S	Q	G	R	E	H	R	Yes	S	Yes	S	E	ESEV
A/duck/Bangladesh/44456/2020	E	D	K	No	RERRRKR↓G	Yes	E	S	Q	G	R	E	H	R	Yes	S	Yes	S	E	ESEV
A/duck/Bangladesh/44469/2020	E	D	K	No	RERRRKR↓G	Yes	E	S	Q	G	R	E	H	R	Yes	S	Yes	S	E	ESEV
A/duck/Bangladesh/44471/2020	E	D	K	No	RERRRKR↓G	Yes	E	S	Q	G	R	E	H	R	Yes	S	Yes	S	E	ESEV
A/duck/Bangladesh/44477/2020	E	D	K	No	RERRRKR↓G	Yes	E	S	Q	G	R	E	H	R	Yes	S	Yes	S	E	ESEV
A/duck/Bangladesh/44484/2020	NA	NA	NA	NA	RERRRKR↓G	Yes	E	S	Q	G	R	E	H	R	Yes	S	Yes	S	E	ESEV
A/duck/Bangladesh/44500/2020	E	D	K	No	RERRRKR↓G	Yes	E	S	Q	G	R	E	H	R	Yes	S	Yes	NA	NA	NA
A/duck/Bangladesh/44502/2020	E	D	K	No	RERRRKR↓G	Yes	E	S	Q	G	R	E	H	R	Yes	S	Yes	S	E	ESEV
A/duck/Bangladesh/44504/2020	E	D	K	No	RERRRKR↓G	Yes	E	S	Q	G	R	E	H	R	Yes	S	Yes	S	E	ESEV
A/duck/Bangladesh/44508/2020	E	D	K	No	RERRRKR↓G	Yes	E	S	Q	G	R	E	H	R	Yes	S	Yes	S	E	ESEV
A/duck/Bangladesh/44523/2020	E	D	K	No	RERRRKR↓G	Yes	E	S	Q	G	R	E	H	R	Yes	S	Yes	S	E	ESEV
A/duck/Bangladesh/44522/2020	E	D	K	No	RERRRKR↓G	Yes	E	S	Q	G	R	E	H	R	Yes	S	Yes	S	E	ESEV
A/Guangxi/31906/2018‡	K	D	K	No	RERRRKR↓G	Yes	E	D	Q	G	R	E	H	R	Yes	S	Yes	S	E	ESEV
A/Jiangsu/32888/2018‡	E	D	K	No	RERRRKR↓G	Yes	E	K	Q	G	G	E	H	R	Yes	S	Yes	S	E	ESEI
A/Guangxi/32797/2018‡	K	D	K	No	RERRRKR↓G	Yes	E	D	Q	G	H	E	H	R	Yes	S	Yes	S	E	ESEV
A/Guangdong/18SF020/2018–09–30‡	V	D	K	No	RERRRKR↓G	Yes	E	D	Q	G	R	E	H	R	Yes	S	Yes	S	E	ESEV
A/Chongqing/00013/2021‡	E	D	K	No	RERRRKR↓G	Yes	E	V	Q	G	R	E	H	R	Yes	S	Yes	S	E	KSEV

	PB2			PB1-F2	HA							NA				M2	NS			
							Receptor binding													
Viruses	E627K	D701N	K482R	Expression	Cleavage site	HA del. 133*	E190D	N193K	Q226L	G228S	S227N/R	E119D†	H274Y	R292K	Stalk del.	S31N	NS del. 80–84	P42S	D92E	PDZ motif
A/Anhui/2021–00011/2020‡	E	D	K	No	RERRRKR↓G	Yes	E	D	Q	G	R	E	H	R	Yes	S	Yes	S	E	KSEV

*H3 numbering.
†N2 numbering.
‡ Human isolates of HPAI A(H5N6) clade 2.3.4.4h.

Appendix Table 3. Hemagglutination inhibition assay of HPAI A(H5N6) viruses clade 2.3.4.4h from Bangladesh

			Polyclonal antibodies*							
Antigen	Subtype	Clade	A/Sichuan/ 26221/2014	A/Fujian-Sanyuan/ 21099/2017	A/gyrfalcon/ Washington/ 41088–6/2014	A/Hubei/ 29578/ 2016	A/duck/ Hyogo/1/ 2016 yogo	A/chicken/ Vietnam/NCV D-15A59/ 2015 15A59	A/duck/ Bangladesh/ 43127/2020‡	A/Northern pintail/ Washington/ 40964/2014
Reference antigen										
A/Sichuan/26221/2014 EPI_ISL_163493†	H5N6	2.3.4.4a	**160**	80	160	<10	320	20	20	1280
A/Fujian-Sanyuan/21099/2017 x PR8 (CNIC-21099) EPI_ISL_341294	H5N6	2.3.4.4b	80	**80**	160	<10	160	10	10	1280
A/gyrfalcon/Washington/41088–6/2014 EPI_ISL_173878	H5N8	2.3.4.4c	160	80	**160**	<10	160	10	10	1280
A/Hubei/29578/2016 x PR8 (CNIC-HB29578) EPI_ISL_341293	H5N6	2.3.4.4d	<10	<10	<10	**80**	<10	<10	20	<10
A/duck/Hyogo/1/2016 EPI_ISL_239351	H5N6	2.3.4.4e	160	80	160	<10	**320**	10	20	1280
A/chicken/Vietnam/NCVD-15A59/2015 EPI_ISL_244518	H5N6	2.3.4.4f	40	20	80	<10	320	**40**	10	640
A/duck/Bangladesh/43127/2020	H5N6	2.3.4.4h	<10	<10	<10	<10	<10	<10	**40**	<10
A/Northern pintail/Washington/40964/2014 EPI_ISL_173877	H5N2	2.3.4.4	80	40	80	<10	160	10	10	**640**
A/Guangdong/18SF020/2018 EPI_ISL_337274	H5N6	2.3.4.4h	<10	<10	<10	10	<10	<10	80	<10
Test antigen										
A/duck/Bangladesh/43129/2020	H5N6	2.3.4.4h	<10	<10	<10	<10	<10	<10	160	<10
A/Common pochard/Bangladesh/42386/2020	H5N6	2.3.4.4h	<10	<10	<10	<10	<10	<10	40	<10
A/environment/Bangladesh/42410/2020	H5N6	2.3.4.4h	<10	<10	<10	<10	<10	<10	80	<10
A/duck/Bangladesh/44424/2020	H5N6	2.3.4.4h	<10	<10	<10	<10	<10	<10	40	<10
A/duck/Bangladesh/44434/2020	H5N6	2.3.4.4h	<10	<10	<10	<10	<10	<10	40	<10
A/duck/Bangladesh/44469/2020	H5N6	2.3.4.4h	<10	<10	<10	<10	<10	<10	80	<10
A/duck/Bangladesh/44477/2020	H5N6	2.3.4.4h	<10	<10	<10	<10	<10	<10	40	<10
A/duck/Bangladesh/44484/2020	H5N6	2.3.4.4h	<10	<10	<10	<10	<10	<10	160	<10
A/duck/Bangladesh/44502/2020	H5N6	2.3.4.4h	<10	<10	<10	<10	<10	<10	40	<10

*Polyclonal antibodies were produced in the ferret. Homologous titers are in bold.
†GISAID accession number.
‡The antiserum against A/duck/Bangladesh/43127/2020 (H5N6) was generated for this study.

A

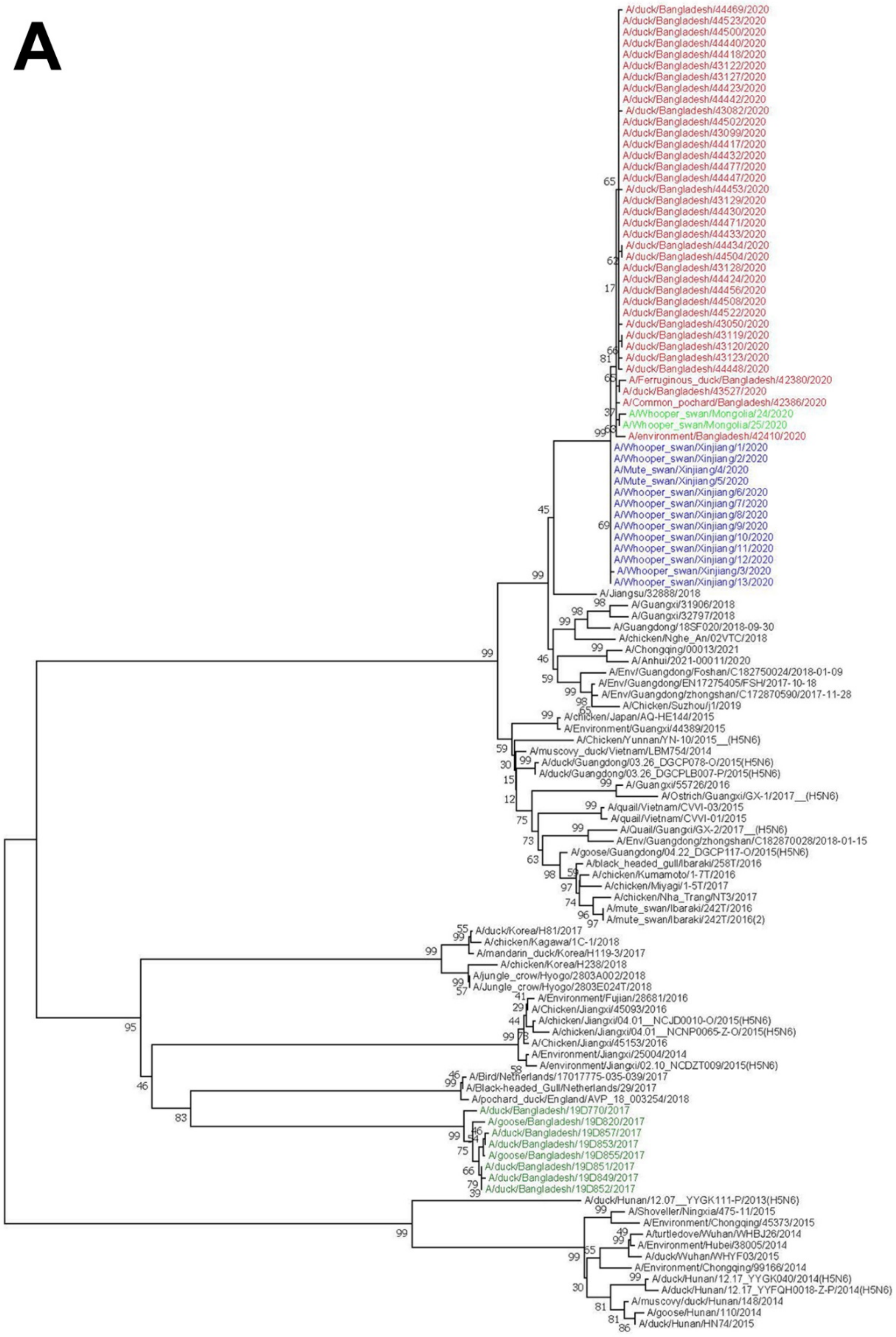

A/duck/Bangladesh/44469/2020
A/duck/Bangladesh/44523/2020
A/duck/Bangladesh/44500/2020
A/duck/Bangladesh/44440/2020
A/duck/Bangladesh/44418/2020
A/duck/Bangladesh/43122/2020
A/duck/Bangladesh/43127/2020
A/duck/Bangladesh/44423/2020
A/duck/Bangladesh/44442/2020
A/duck/Bangladesh/43082/2020
A/duck/Bangladesh/44502/2020
A/duck/Bangladesh/43099/2020
A/duck/Bangladesh/44417/2020
A/duck/Bangladesh/44432/2020
A/duck/Bangladesh/44477/2020
A/duck/Bangladesh/44447/2020
A/duck/Bangladesh/44453/2020
A/duck/Bangladesh/43129/2020
A/duck/Bangladesh/44430/2020
A/duck/Bangladesh/44471/2020
A/duck/Bangladesh/44433/2020
A/duck/Bangladesh/44434/2020
A/duck/Bangladesh/44504/2020
A/duck/Bangladesh/43128/2020
A/duck/Bangladesh/44424/2020
A/duck/Bangladesh/44456/2020
A/duck/Bangladesh/44508/2020
A/duck/Bangladesh/44522/2020
A/duck/Bangladesh/43050/2020
A/duck/Bangladesh/43119/2020
A/duck/Bangladesh/43120/2020
A/duck/Bangladesh/43123/2020
A/duck/Bangladesh/44448/2020
A/Ferruginous_duck/Bangladesh/42380/2020
A/duck/Bangladesh/43527/2020
A/Common_pochard/Bangladesh/42386/2020
A/Whooper_swan/Mongolia/24/2020
A/Whooper_swan/Mongolia/25/2020
A/environment/Bangladesh/42410/2020
A/Whooper_swan/Xinjiang/1/2020
A/Whooper_swan/Xinjiang/2/2020
A/Mute_swan/Xinjiang/4/2020
A/Mute_swan/Xinjiang/5/2020
A/Whooper_swan/Xinjiang/6/2020
A/Whooper_swan/Xinjiang/7/2020
A/Whooper_swan/Xinjiang/8/2020
A/Whooper_swan/Xinjiang/9/2020
A/Whooper_swan/Xinjiang/10/2020
A/Whooper_swan/Xinjiang/11/2020
A/Whooper_swan/Xinjiang/12/2020
A/Whooper_swan/Xinjiang/3/2020
A/Whooper_swan/Xinjiang/13/2020
A/Jiangsu/32888/2018
A/Guangxi/31906/2018
A/Guangxi/32797/2018
A/Guangdong/18SF020/2018-09-30
A/chicken/Nghe_An/02VTC/2018
A/Chongqing/00013/2021
A/Anhui/2021-00011/2020
A/Env/Guangdong/Foshan/C182750024/2018-01-09
A/Env/Guangdong/EN17275405/FSH/2017-10-18
A/Env/Guangdong/zhongshan/C172870590/2017-11-28
A/Chicken/Suzhou/j1/2019
A/chicken/Japan/AQ-HE144/2015
A/Environment/Guangxi/44389/2015
A/Chicken/Yunnan/YN-10/2015__(H5N6)
A/muscovy_duck/Vietnam/LBM754/2014
A/duck/Guangdong/03.26_DGCP078-O/2015(H5N6)
A/duck/Guangdong/03.26_DGCPLB007-P/2015(H5N6)
A/Guangxi/55726/2016
A/Ostrich/Guangxi/GX-1/2017__(H5N6)
A/quail/Vietnam/CVVI-03/2015
A/quail/Vietnam/CVVI-01/2015
A/Quail/Guangxi/GX-2/2017__(H5N6)
A/Env/Guangdong/zhongshan/C182870028/2018-01-15
A/goose/Guangdong/04.22_DGCP117-O/2015(H5N6)
A/black_headed_gull/Ibaraki/258T/2016
A/chicken/Kumamoto/1-7T/2016
A/chicken/Miyagi/1-5T/2017
A/chicken/Nha_Trang/NT3/2017
A/mute_swan/Ibaraki/242T/2016
A/mute_swan/Ibaraki/242T/2016(2)
A/duck/Korea/H81/2017
A/chicken/Kagawa/1C-1/2018
A/mandarin_duck/Korea/H119-3/2017
A/chicken/Korea/H238/2018
A/jungle_crow/Hyogo/2803A002/2018
A/Jungle_crow/Hyogo/2803E024T/2018
A/Environment/Fujian/28681/2016
A/Chicken/Jiangxi/45093/2016
A/chicken/Jiangxi/04.01__NCJD0010-O/2015(H5N6)
A/chicken/Jiangxi/04.01__NCNP0065-Z-O/2015(H5N6)
A/Chicken/Jiangxi/45153/2016
A/Environment/Jiangxi/25004/2014
A/environment/Jiangxi/02.10_NCDZT009/2015(H5N6)
A/Bird/Netherlands/17017775-035-039/2017
A/Black-headed_Gull/Netherlands/29/2017
A/pochard_duck/England/AVP_18_003254/2018
A/duck/Bangladesh/19D770/2017
A/goose/Bangladesh/19D820/2017
A/duck/Bangladesh/19D857/2017
A/duck/Bangladesh/19D853/2017
A/goose/Bangladesh/19D855/2017
A/duck/Bangladesh/19D851/2017
A/duck/Bangladesh/19D849/2017
A/duck/Bangladesh/19D852/2017
A/duck/Hunan/12.07__YYGK111-P/2013(H5N6)
A/Shoveller/Ningxia/475-11/2015
A/Environment/Chongqing/45373/2015
A/turtledove/Wuhan/WHBJ26/2014
A/Environment/Hubei/38005/2014
A/duck/Wuhan/WHYF03/2015
A/Environment/Chongqing/99166/2014
A/duck/Hunan/12.17_YYGK040/2014(H5N6)
A/duck/Hunan/12.17_YYFQH0018-Z-P/2014(H5N6)
A/muscovy/duck/Hunan/148/2014
A/goose/Hunan/110/2014
A/duck/Hunan/HN74/2015

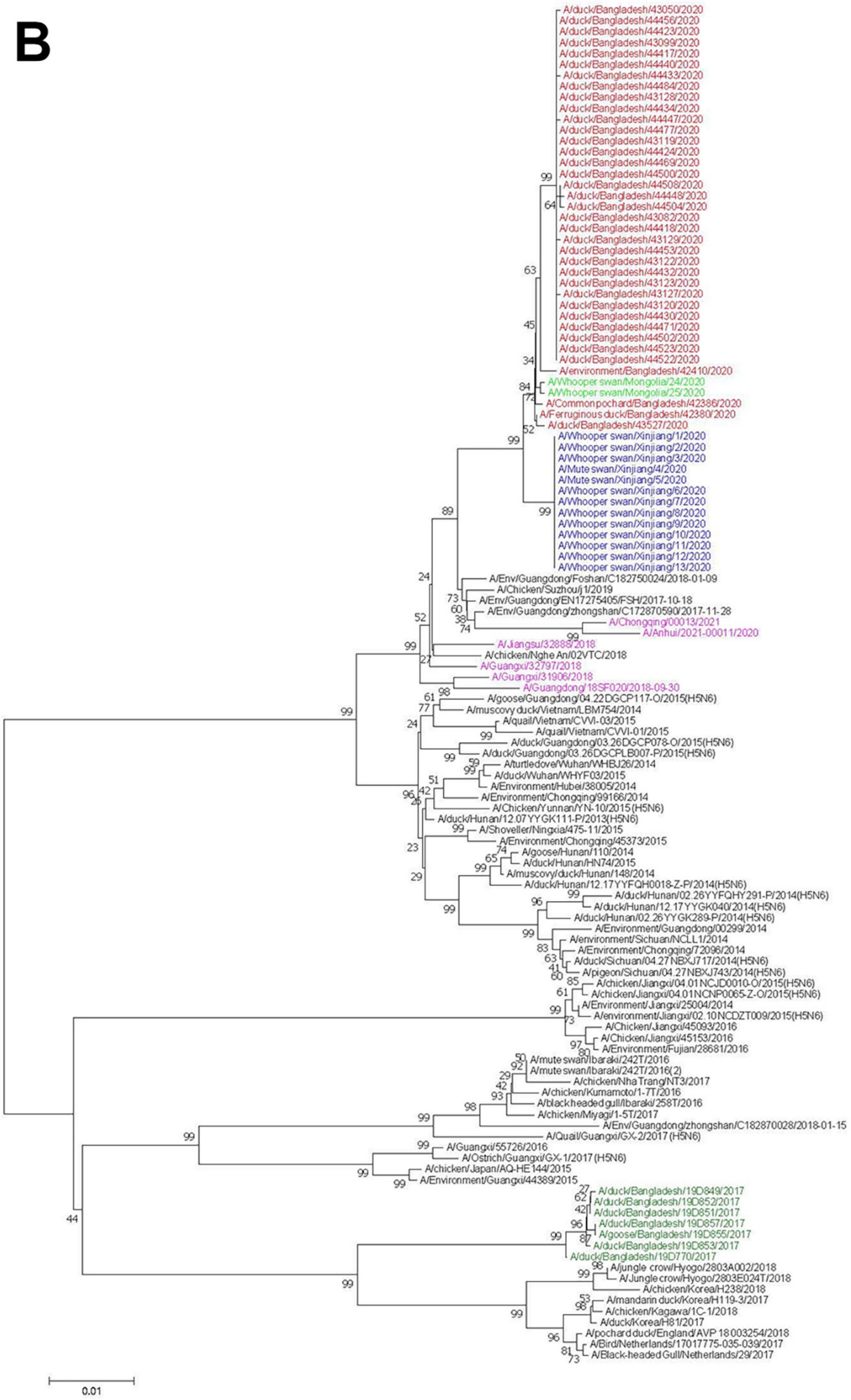
B
A/duck/Bangladesh/43050/2020
A/duck/Bangladesh/44456/2020
A/duck/Bangladesh/44423/2020
A/duck/Bangladesh/43099/2020
A/duck/Bangladesh/44417/2020
A/duck/Bangladesh/44440/2020
A/duck/Bangladesh/44433/2020
A/duck/Bangladesh/44484/2020
A/duck/Bangladesh/43128/2020
A/duck/Bangladesh/44434/2020
A/duck/Bangladesh/44447/2020
A/duck/Bangladesh/44477/2020
A/duck/Bangladesh/43119/2020
A/duck/Bangladesh/44424/2020
A/duck/Bangladesh/44469/2020
A/duck/Bangladesh/44500/2020
A/duck/Bangladesh/44508/2020
A/duck/Bangladesh/44448/2020
A/duck/Bangladesh/44504/2020
A/duck/Bangladesh/43082/2020
A/duck/Bangladesh/44418/2020
A/duck/Bangladesh/43129/2020
A/duck/Bangladesh/44453/2020
A/duck/Bangladesh/43122/2020
A/duck/Bangladesh/44432/2020
A/duck/Bangladesh/43123/2020
A/duck/Bangladesh/43127/2020
A/duck/Bangladesh/43120/2020
A/duck/Bangladesh/44430/2020
A/duck/Bangladesh/44471/2020
A/duck/Bangladesh/44502/2020
A/duck/Bangladesh/44523/2020
A/duck/Bangladesh/44522/2020
A/environment/Bangladesh/42410/2020
A/Whooper swan/Mongolia/24/2020
A/Whooper swan/Mongolia/25/2020
A/Common pochard/Bangladesh/42386/2020
A/Ferruginous duck/Bangladesh/42380/2020
A/duck/Bangladesh/43527/2020
A/Whooper swan/Xinjiang/1/2020
A/Whooper swan/Xinjiang/2/2020
A/Whooper swan/Xinjiang/3/2020
A/Mute swan/Xinjiang/4/2020
A/Mute swan/Xinjiang/5/2020
A/Whooper swan/Xinjiang/6/2020
A/Whooper swan/Xinjiang/7/2020
A/Whooper swan/Xinjiang/8/2020
A/Whooper swan/Xinjiang/9/2020
A/Whooper swan/Xinjiang/10/2020
A/Whooper swan/Xinjiang/11/2020
A/Whooper swan/Xinjiang/12/2020
A/Whooper swan/Xinjiang/13/2020
A/Env/Guangdong/Foshan/C182750024/2018-01-09
A/Chicken/Suzhou/j1/2019
A/Env/Guangdong/EN17275405/FSH/2017-10-18
A/Env/Guangdong/zhongshan/C172870590/2017-11-28
A/Chongqing/00013/2021
A/Anhui/2021-00011/2020
A/Jiangsu/32888/2018
A/chicken/Nghe An/02VTC/2018
A/Guangxi/32797/2018
A/Guangxi/31906/2018
A/Guangdong/18SF020/2018-09-30
A/goose/Guangdong/04.22DGCP117-O/2015(H5N6)
A/muscovy duck/Vietnam/LBM754/2014
A/quail/Vietnam/CVVI-03/2015
A/quail/Vietnam/CVVI-01/2015
A/duck/Guangdong/03.26DGCP078-O/2015(H5N6)
A/duck/Guangdong/03.26DGCPLB007-P/2015(H5N6)
A/turtledove/Wuhan/WHBJ26/2014
A/duck/Wuhan/WHYF03/2015
A/Environment/Hubei/38005/2014
A/Environment/Chongqing/99166/2014
A/Chicken/Yunnan/YN-10/2015(H5N6)
A/duck/Hunan/12.07YYGK111-P/2013(H5N6)
A/Shoveller/Ningxia/475-11/2015
A/Environment/Chongqing/45373/2015
A/goose/Hunan/110/2014
A/duck/Hunan/HN74/2015
A/muscovy/duck/Hunan/148/2014
A/duck/Hunan/12.17YYFQH0018-Z-P/2014(H5N6)
A/duck/Hunan/02.26YYFQHY291-P/2014(H5N6)
A/duck/Hunan/12.17YYGK040/2014(H5N6)
A/duck/Hunan/02.26YYGK289-P/2014(H5N6)
A/Environment/Guangdong/00299/2014
A/environment/Sichuan/NCLL1/2014
A/Environment/Chongqing/72096/2014
A/duck/Sichuan/04.27NBXJ717/2014(H5N6)
A/pigeon/Sichuan/04.27NBXJ743/2014(H5N6)
A/chicken/Jiangxi/04.01NCJD0010-O/2015(H5N6)
A/chicken/Jiangxi/04.01NCNP0065-Z-O/2015(H5N6)
A/Environment/Jiangxi/25004/2014
A/environment/Jiangxi/02.10NCDZT009/2015(H5N6)
A/Chicken/Jiangxi/45093/2016
A/Chicken/Jiangxi/45153/2016
A/Environment/Fujian/28681/2016
A/mute swan/Ibaraki/242T/2016
A/mute swan/Ibaraki/242T/2016(2)
A/chicken/Nha Trang/NT3/2017
A/chicken/Kumamoto/1-7T/2016
A/black headed gull/Ibaraki/258T/2016
A/chicken/Miyagi/1-5T/2017
A/Env/Guangdong/zhongshan/C182870028/2018-01-15
A/Quail/Guangxi/GX-2/2017(H5N6)
A/Guangxi/55726/2016
A/Ostrich/Guangxi/GX-1/2017(H5N6)
A/chicken/Japan/AQ-HE144/2015
A/Environment/Guangxi/44389/2015
A/duck/Bangladesh/19D849/2017
A/duck/Bangladesh/19D852/2017
A/duck/Bangladesh/19D851/2017
A/duck/Bangladesh/19D857/2017
A/goose/Bangladesh/19D855/2017
A/duck/Bangladesh/19D853/2017
A/duck/Bangladesh/19D770/2017
A/jungle crow/Hyogo/2803A002/2018
A/Jungle crow/Hyogo/2803E024T/2018
A/chicken/Korea/H238/2018
A/mandarin duck/Korea/H119-3/2017
A/chicken/Kagawa/1C-1/2018
A/duck/Korea/H81/2017
A/pochard duck/England/AVP 18003254/2018
A/Bird/Netherlands/17017775-035-039/2017
A/Black-headed Gull/Netherlands/29/2017
0.01

C

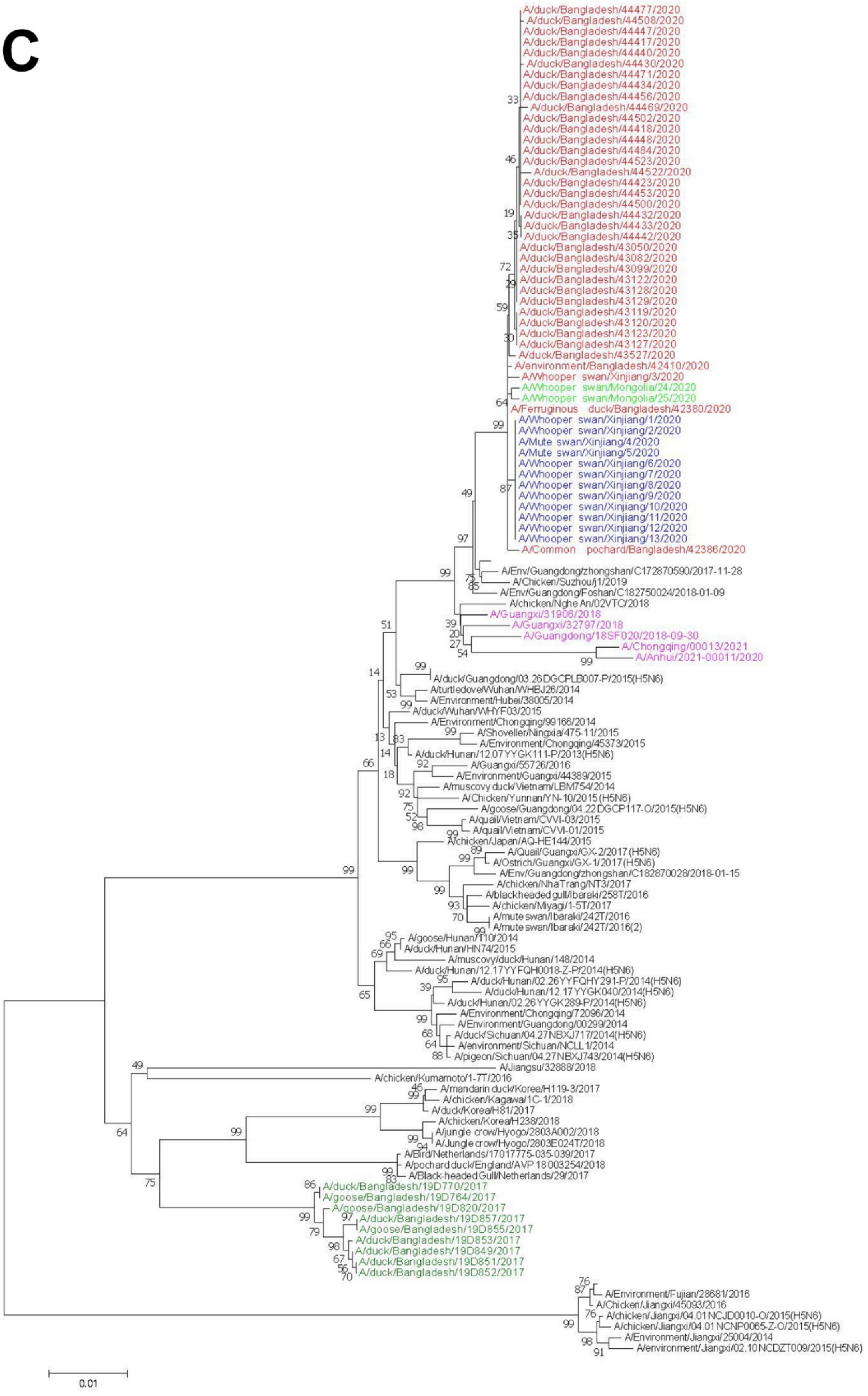

A/duck/Bangladesh/44477/2020
A/duck/Bangladesh/44508/2020
A/duck/Bangladesh/44447/2020
A/duck/Bangladesh/44417/2020
A/duck/Bangladesh/44440/2020
A/duck/Bangladesh/44430/2020
A/duck/Bangladesh/44471/2020
A/duck/Bangladesh/44434/2020
A/duck/Bangladesh/44456/2020
A/duck/Bangladesh/44469/2020
A/duck/Bangladesh/44502/2020
A/duck/Bangladesh/44418/2020
A/duck/Bangladesh/44448/2020
A/duck/Bangladesh/44484/2020
A/duck/Bangladesh/44523/2020
A/duck/Bangladesh/44522/2020
A/duck/Bangladesh/44423/2020
A/duck/Bangladesh/44453/2020
A/duck/Bangladesh/44500/2020
A/duck/Bangladesh/44432/2020
A/duck/Bangladesh/44433/2020
A/duck/Bangladesh/44442/2020
A/duck/Bangladesh/43050/2020
A/duck/Bangladesh/43082/2020
A/duck/Bangladesh/43099/2020
A/duck/Bangladesh/43122/2020
A/duck/Bangladesh/43128/2020
A/duck/Bangladesh/43129/2020
A/duck/Bangladesh/43119/2020
A/duck/Bangladesh/43120/2020
A/duck/Bangladesh/43123/2020
A/duck/Bangladesh/43127/2020
A/duck/Bangladesh/43527/2020
A/environment/Bangladesh/42410/2020
A/Whooper swan/Xinjiang/3/2020
A/Whooper swan/Mongolia/24/2020
A/Whooper swan/Mongolia/25/2020
A/Ferruginous duck/Bangladesh/42380/2020
A/Whooper swan/Xinjiang/1/2020
A/Whooper swan/Xinjiang/2/2020
A/Mute swan/Xinjiang/4/2020
A/Mute swan/Xinjiang/5/2020
A/Whooper swan/Xinjiang/6/2020
A/Whooper swan/Xinjiang/7/2020
A/Whooper swan/Xinjiang/8/2020
A/Whooper swan/Xinjiang/9/2020
A/Whooper swan/Xinjiang/10/2020
A/Whooper swan/Xinjiang/11/2020
A/Whooper swan/Xinjiang/12/2020
A/Whooper swan/Xinjiang/13/2020
A/Common pochard/Bangladesh/42386/2020
A/Env/Guangdong/zhongshan/C172870590/2017-11-28
A/Chicken/Suzhou/j1/2019
A/Env/Guangdong/Foshan/C182750024/2018-01-09
A/chicken/Nghe An/02VTC/2018
A/Guangxi/31906/2018
A/Guangxi/32797/2018
A/Guangdong/18SF020/2018-09-30
A/Chongqing/00013/2021
A/Anhui/2021-00011/2020
A/duck/Guangdong/03.26DGCPLB007-P/2015(H5N6)
A/turtledove/Wuhan/WHBJ26/2014
A/Environment/Hubei/38005/2014
A/duck/Wuhan/WHYF03/2015
A/Environment/Chongqing/99166/2014
A/Shoveller/Ningxia/475-11/2015
A/Environment/Chongqing/45373/2015
A/duck/Hunan/12.07YYGK111-P/2013(H5N6)
A/Guangxi/55726/2016
A/Environment/Guangxi/44389/2015
A/muscovy duck/Vietnam/LBM754/2014
A/Chicken/Yunnan/YN-10/2015(H5N6)
A/goose/Guangdong/04.22DGCP117-O/2015(H5N6)
A/quail/Vietnam/CVVI-03/2015
A/quail/Vietnam/CVVI-01/2015
A/chicken/Japan/AQ-HE144/2015
A/Quail/Guangxi/GX-2/2017(H5N6)
A/Ostrich/Guangxi/GX-1/2017(H5N6)
A/Env/Guangdong/zhongshan/C182870028/2018-01-15
A/chicken/NhaTrang/NT3/2017
A/blackheaded gull/Ibaraki/258T/2016
A/chicken/Miyagi/1-5T/2017
A/mute swan/Ibaraki/242T/2016
A/mute swan/Ibaraki/242T/2016(2)
A/goose/Hunan/110/2014
A/duck/Hunan/HN74/2015
A/muscovy/duck/Hunan/148/2014
A/duck/Hunan/12.17YYFQH0018-Z-P/2014(H5N6)
A/duck/Hunan/02.26YYFQHY291-P/2014(H5N6)
A/duck/Hunan/12.17YYGK040/2014(H5N6)
A/duck/Hunan/02.26YYGK289-P/2014(H5N6)
A/Environment/Chongqing/72096/2014
A/Environment/Guangdong/00299/2014
A/duck/Sichuan/04.27NBXJ717/2014(H5N6)
A/environment/Sichuan/NCLL1/2014
A/pigeon/Sichuan/04.27NBXJ743/2014(H5N6)
A/Jiangsu/32888/2018
A/chicken/Kumamoto/1-7T/2016
A/mandarin duck/Korea/H119-3/2017
A/chicken/Kagawa/1C-1/2018
A/duck/Korea/H81/2017
A/chicken/Korea/H238/2018
A/jungle crow/Hyogo/2803A002/2018
A/Jungle crow/Hyogo/2803E024T/2018
A/Bird/Netherlands/17017775-035-039/2017
A/pochard duck/England/AVP 18003254/2018
A/Black-headed Gull/Netherlands/29/2017
A/duck/Bangladesh/19D770/2017
A/goose/Bangladesh/19D764/2017
A/goose/Bangladesh/19D820/2017
A/duck/Bangladesh/19D857/2017
A/goose/Bangladesh/19D855/2017
A/duck/Bangladesh/19D853/2017
A/duck/Bangladesh/19D849/2017
A/duck/Bangladesh/19D851/2017
A/duck/Bangladesh/19D852/2017
A/Environment/Fujian/28681/2016
A/Chicken/Jiangxi/45093/2016
A/chicken/Jiangxi/04.01NCJD0010-O/2015(H5N6)
A/chicken/Jiangxi/04.01NCNP0065-Z-O/2015(H5N6)
A/Environment/Jiangxi/25004/2014
A/environment/Jiangxi/02.10NCDZT009/2015(H5N6)
0.01

D

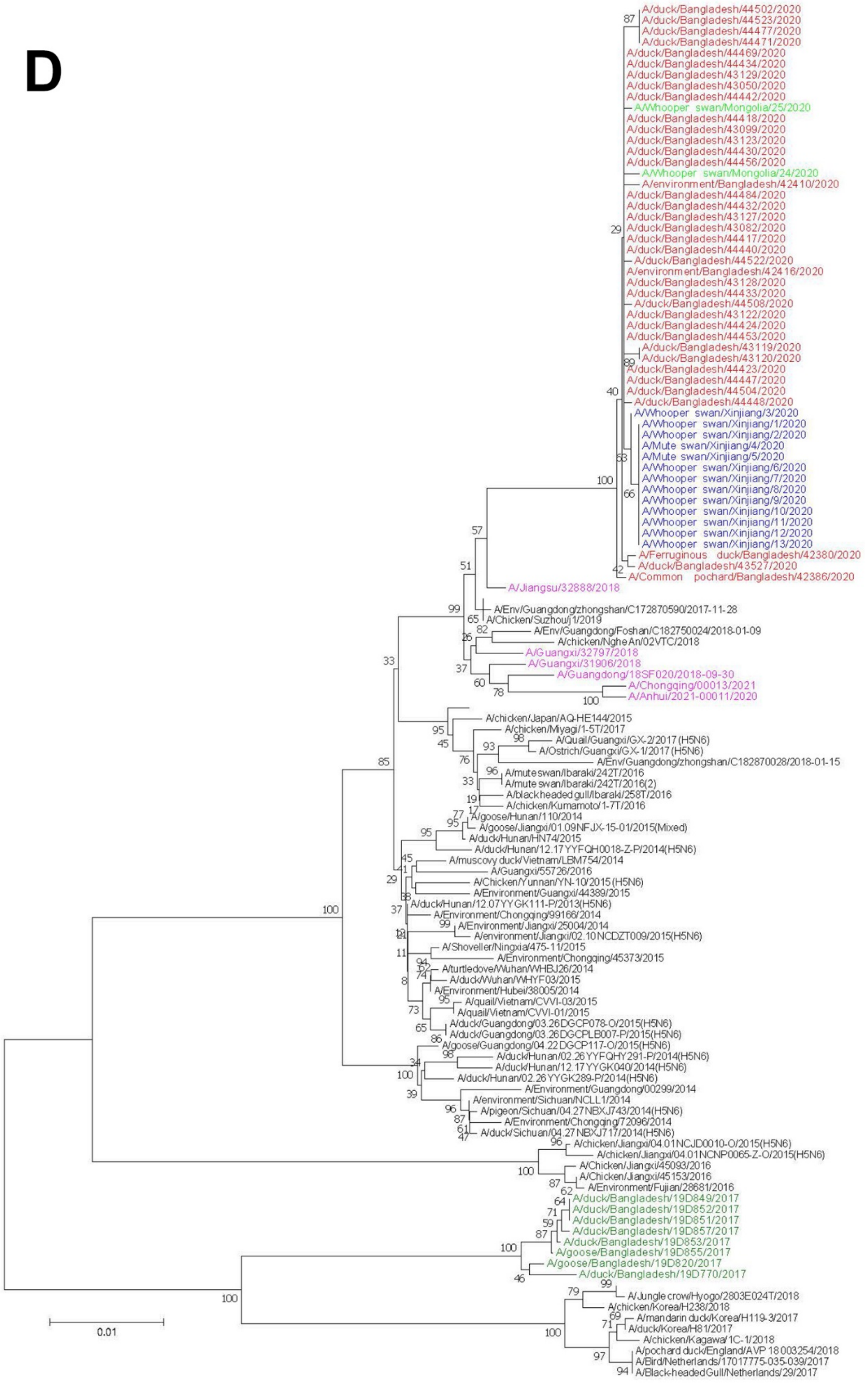
A/duck/Bangladesh/44502/2020
A/duck/Bangladesh/44523/2020
A/duck/Bangladesh/44477/2020
A/duck/Bangladesh/44471/2020
A/duck/Bangladesh/44469/2020
A/duck/Bangladesh/44434/2020
A/duck/Bangladesh/43129/2020
A/duck/Bangladesh/43050/2020
A/duck/Bangladesh/44442/2020
A/Whooper swan/Mongolia/25/2020
A/duck/Bangladesh/44418/2020
A/duck/Bangladesh/43099/2020
A/duck/Bangladesh/43123/2020
A/duck/Bangladesh/44430/2020
A/duck/Bangladesh/44456/2020
A/Whooper swan/Mongolia/24/2020
A/environment/Bangladesh/42410/2020
A/duck/Bangladesh/44484/2020
A/duck/Bangladesh/44432/2020
A/duck/Bangladesh/43127/2020
A/duck/Bangladesh/43082/2020
A/duck/Bangladesh/44417/2020
A/duck/Bangladesh/44440/2020
A/duck/Bangladesh/44522/2020
A/environment/Bangladesh/42416/2020
A/duck/Bangladesh/43128/2020
A/duck/Bangladesh/44433/2020
A/duck/Bangladesh/44508/2020
A/duck/Bangladesh/43122/2020
A/duck/Bangladesh/44424/2020
A/duck/Bangladesh/44453/2020
A/duck/Bangladesh/43119/2020
A/duck/Bangladesh/43120/2020
A/duck/Bangladesh/44423/2020
A/duck/Bangladesh/44447/2020
A/duck/Bangladesh/44504/2020
A/duck/Bangladesh/44448/2020
A/Whooper swan/Xinjiang/3/2020
A/Whooper swan/Xinjiang/1/2020
A/Whooper swan/Xinjiang/2/2020
A/Mute swan/Xinjiang/4/2020
A/Mute swan/Xinjiang/5/2020
A/Whooper swan/Xinjiang/6/2020
A/Whooper swan/Xinjiang/7/2020
A/Whooper swan/Xinjiang/8/2020
A/Whooper swan/Xinjiang/9/2020
A/Whooper swan/Xinjiang/10/2020
A/Whooper swan/Xinjiang/11/2020
A/Whooper swan/Xinjiang/12/2020
A/Whooper swan/Xinjiang/13/2020
A/Ferruginous duck/Bangladesh/42380/2020
A/duck/Bangladesh/43527/2020
A/Common pochard/Bangladesh/42386/2020
A/Jiangsu/32888/2018
A/Env/Guangdong/zhongshan/C172870590/2017-11-28
A/Chicken/Suzhou/j1/2019
A/Env/Guangdong/Foshan/C182750024/2018-01-09
A/chicken/Nghe An/02VTC/2018
A/Guangxi/32797/2018
A/Guangxi/31906/2018
A/Guangdong/18SF020/2018-09-30
A/Chongqing/00013/2021
A/Anhui/2021-00011/2020
A/chicken/Japan/AQ-HE144/2015
A/chicken/Miyagi/1-5T/2017
A/Quail/Guangxi/GX-2/2017(H5N6)
A/Ostrich/Guangxi/GX-1/2017(H5N6)
A/Env/Guangdong/zhongshan/C182870028/2018-01-15
A/mute swan/Ibaraki/242T/2016
A/mute swan/Ibaraki/242T/2016(2)
A/black headed gull/Ibaraki/258T/2016
A/chicken/Kumamoto/1-7T/2016
A/goose/Hunan/110/2014
A/goose/Jiangxi/01.09NFJX-15-01/2015(Mixed)
A/duck/Hunan/HN74/2015
A/duck/Hunan/12.17YYFQH0018-Z-P/2014(H5N6)
A/muscovy duck/Vietnam/LBM754/2014
A/Guangxi/55726/2016
A/Chicken/Yunnan/YN-10/2015(H5N6)
A/Environment/Guangxi/44389/2015
A/duck/Hunan/12.07YYGK111-P/2013(H5N6)
A/Environment/Chongqing/99166/2014
A/Environment/Jiangxi/25004/2014
A/environment/Jiangxi/02.10NCDZT009/2015(H5N6)
A/Shoveller/Ningxia/475-11/2015
A/Environment/Chongqing/45373/2015
A/turtledove/Wuhan/WHBJ26/2014
A/duck/Wuhan/WHYF03/2015
A/Environment/Hubei/38005/2014
A/quail/Vietnam/CVVI-03/2015
A/quail/Vietnam/CVVI-01/2015
A/duck/Guangdong/03.26DGCP078-O/2015(H5N6)
A/duck/Guangdong/03.26DGCPLB007-P/2015(H5N6)
A/goose/Guangdong/04.22DGCP117-O/2015(H5N6)
A/duck/Hunan/02.26YYFQHY291-P/2014(H5N6)
A/duck/Hunan/12.17YYGK040/2014(H5N6)
A/duck/Hunan/02.26YYGK289-P/2014(H5N6)
A/Environment/Guangdong/00299/2014
A/environment/Sichuan/NCLL1/2014
A/pigeon/Sichuan/04.27NBXJ743/2014(H5N6)
A/Environment/Chongqing/72096/2014
A/duck/Sichuan/04.27NBXJ717/2014(H5N6)
A/chicken/Jiangxi/04.01NCJD0010-O/2015(H5N6)
A/chicken/Jiangxi/04.01NCNP0065-Z-O/2015(H5N6)
A/Chicken/Jiangxi/45093/2016
A/Chicken/Jiangxi/45153/2016
A/Environment/Fujian/28681/2016
A/duck/Bangladesh/19D849/2017
A/duck/Bangladesh/19D852/2017
A/duck/Bangladesh/19D851/2017
A/duck/Bangladesh/19D857/2017
A/duck/Bangladesh/19D853/2017
A/goose/Bangladesh/19D855/2017
A/goose/Bangladesh/19D820/2017
A/duck/Bangladesh/19D770/2017
A/Jungle crow/Hyogo/2803E024T/2018
A/chicken/Korea/H238/2018
A/mandarin duck/Korea/H119-3/2017
A/duck/Korea/H81/2017
A/chicken/Kagawa/1C-1/2018
A/pochard duck/England/AVP 18003254/2018
A/Bird/Netherlands/17017775-035-039/2017
A/Black-headed Gull/Netherlands/29/2017
0.01

E

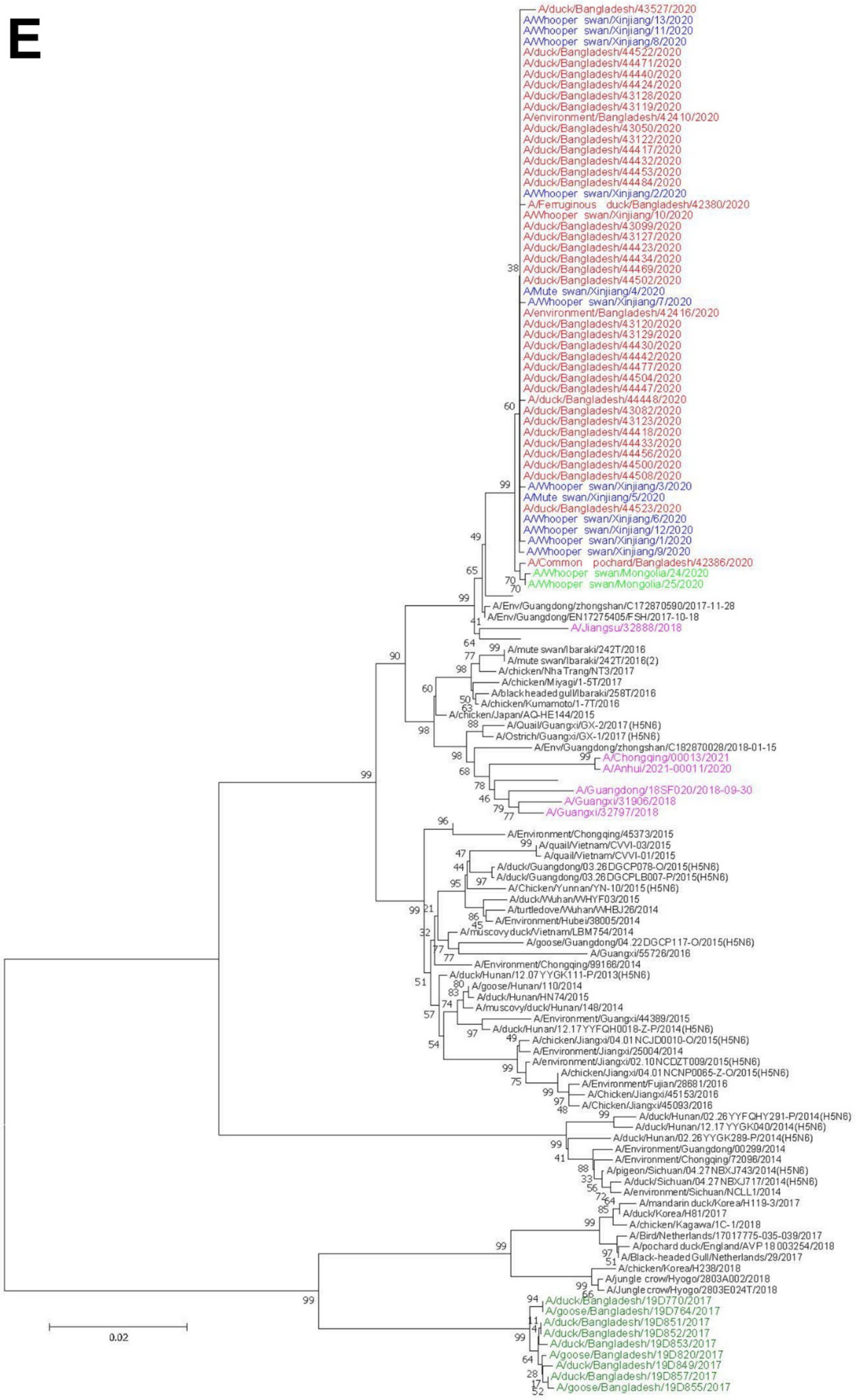
A/duck/Bangladesh/43527/2020
A/Whooper swan/Xinjiang/13/2020
A/Whooper swan/Xinjiang/11/2020
A/Whooper swan/Xinjiang/8/2020
A/duck/Bangladesh/44522/2020
A/duck/Bangladesh/44471/2020
A/duck/Bangladesh/44440/2020
A/duck/Bangladesh/44424/2020
A/duck/Bangladesh/43128/2020
A/duck/Bangladesh/43119/2020
A/environment/Bangladesh/42410/2020
A/duck/Bangladesh/43050/2020
A/duck/Bangladesh/43122/2020
A/duck/Bangladesh/44417/2020
A/duck/Bangladesh/44432/2020
A/duck/Bangladesh/44453/2020
A/duck/Bangladesh/44484/2020
A/Whooper swan/Xinjiang/2/2020
A/Ferruginous duck/Bangladesh/42380/2020
A/Whooper swan/Xinjiang/10/2020
A/duck/Bangladesh/43099/2020
A/duck/Bangladesh/43127/2020
A/duck/Bangladesh/44423/2020
A/duck/Bangladesh/44434/2020
A/duck/Bangladesh/44469/2020
A/duck/Bangladesh/44502/2020
A/Mute swan/Xinjiang/4/2020
A/Whooper swan/Xinjiang/7/2020
A/environment/Bangladesh/42416/2020
A/duck/Bangladesh/43120/2020
A/duck/Bangladesh/43129/2020
A/duck/Bangladesh/44430/2020
A/duck/Bangladesh/44442/2020
A/duck/Bangladesh/44477/2020
A/duck/Bangladesh/44504/2020
A/duck/Bangladesh/44447/2020
A/duck/Bangladesh/44448/2020
A/duck/Bangladesh/43082/2020
A/duck/Bangladesh/43123/2020
A/duck/Bangladesh/44418/2020
A/duck/Bangladesh/44433/2020
A/duck/Bangladesh/44456/2020
A/duck/Bangladesh/44500/2020
A/duck/Bangladesh/44508/2020
A/Whooper swan/Xinjiang/3/2020
A/Mute swan/Xinjiang/5/2020
A/duck/Bangladesh/44523/2020
A/Whooper swan/Xinjiang/6/2020
A/Whooper swan/Xinjiang/12/2020
A/Whooper swan/Xinjiang/1/2020
A/Whooper swan/Xinjiang/9/2020
A/Common pochard/Bangladesh/42386/2020
A/Whooper swan/Mongolia/24/2020
A/Whooper swan/Mongolia/25/2020
A/Env/Guangdong/zhongshan/C172870590/2017-11-28
A/Env/Guangdong/EN17275405/FSH/2017-10-18
A/Jiangsu/32888/2018
A/mute swan/Ibaraki/242T/2016
A/mute swan/Ibaraki/242T/2016(2)
A/chicken/Nha Trang/NT3/2017
A/chicken/Miyagi/1-5T/2017
A/black headed gull/Ibaraki/258T/2016
A/chicken/Kumamoto/1-7T/2016
A/chicken/Japan/AQ-HE144/2015
A/Quail/Guangxi/GX-2/2017 (H5N6)
A/Ostrich/Guangxi/GX-1/2017 (H5N6)
A/Env/Guangdong/zhongshan/C182870028/2018-01-15
A/Chongqing/00013/2021
A/Anhui/2021-00011/2020
A/Guangdong/18SF020/2018-09-30
A/Guangxi/31906/2018
A/Guangxi/32797/2018
A/Environment/Chongqing/45373/2015
A/quail/Vietnam/CVVI-03/2015
A/quail/Vietnam/CVVI-01/2015
A/duck/Guangdong/03.26DGCP078-O/2015(H5N6)
A/duck/Guangdong/03.26DGCPLB007-P/2015(H5N6)
A/Chicken/Yunnan/YN-10/2015(H5N6)
A/duck/Wuhan/WHYF03/2015
A/turtledove/Wuhan/WHBJ26/2014
A/Environment/Hubei/38005/2014
A/muscovy duck/Vietnam/LBM754/2014
A/goose/Guangdong/04.22DGCP117-O/2015(H5N6)
A/Guangxi/55726/2016
A/Environment/Chongqing/99166/2014
A/duck/Hunan/12.07YYGK111-P/2013(H5N6)
A/goose/Hunan/110/2014
A/duck/Hunan/HN74/2015
A/muscovy/duck/Hunan/148/2014
A/Environment/Guangxi/44389/2015
A/duck/Hunan/12.17YYFQH0018-Z-P/2014(H5N6)
A/chicken/Jiangxi/04.01NCJD0010-O/2015(H5N6)
A/Environment/Jiangxi/25004/2014
A/environment/Jiangxi/02.10NCDZT009/2015(H5N6)
A/chicken/Jiangxi/04.01NCNP0065-Z-O/2015(H5N6)
A/Environment/Fujian/28681/2016
A/Chicken/Jiangxi/45153/2016
A/Chicken/Jiangxi/45093/2016
A/duck/Hunan/02.26YYFQHY291-P/2014(H5N6)
A/duck/Hunan/12.17YYGK040/2014(H5N6)
A/duck/Hunan/02.26YYGK289-P/2014(H5N6)
A/Environment/Guangdong/00299/2014
A/Environment/Chongqing/72096/2014
A/pigeon/Sichuan/04.27NBXJ743/2014(H5N6)
A/duck/Sichuan/04.27NBXJ717/2014(H5N6)
A/environment/Sichuan/NCLL1/2014
A/mandarin duck/Korea/H119-3/2017
A/duck/Korea/H81/2017
A/chicken/Kagawa/1C-1/2018
A/Bird/Netherlands/17017775-035-039/2017
A/pochard duck/England/AVP18003254/2018
A/Black-headed Gull/Netherlands/29/2017
A/chicken/Korea/H238/2018
A/jungle crow/Hyogo/2803A002/2018
A/Jungle crow/Hyogo/2803E024T/2018
A/duck/Bangladesh/19D770/2017
A/goose/Bangladesh/19D764/2017
A/duck/Bangladesh/19D851/2017
A/duck/Bangladesh/19D852/2017
A/duck/Bangladesh/19D853/2017
A/goose/Bangladesh/19D820/2017
A/duck/Bangladesh/19D849/2017
A/duck/Bangladesh/19D857/2017
A/goose/Bangladesh/19D855/2017
0.02

F

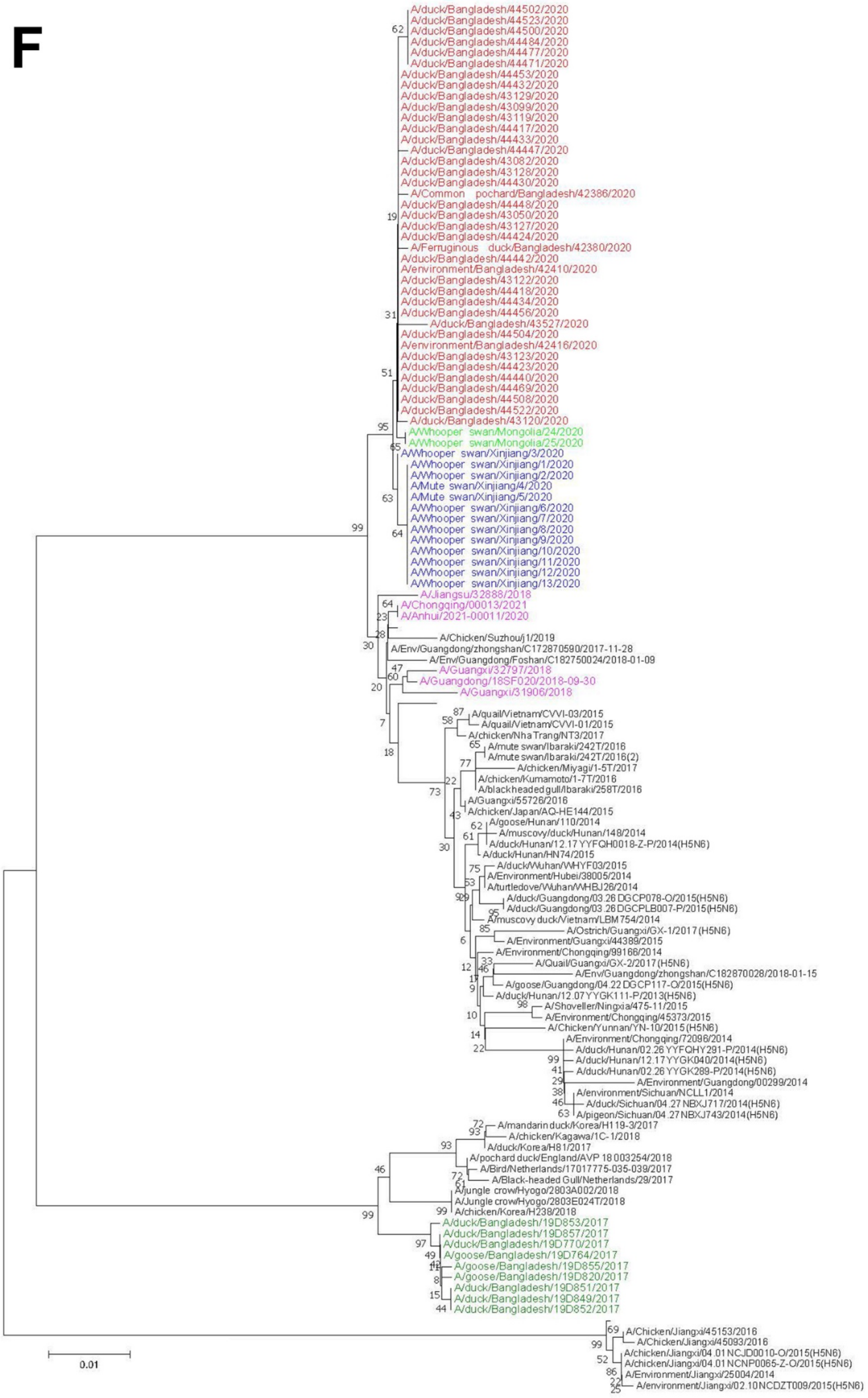
A/duck/Bangladesh/44502/2020
A/duck/Bangladesh/44523/2020
A/duck/Bangladesh/44500/2020
A/duck/Bangladesh/44484/2020
A/duck/Bangladesh/44477/2020
A/duck/Bangladesh/44471/2020
A/duck/Bangladesh/44453/2020
A/duck/Bangladesh/44432/2020
A/duck/Bangladesh/43129/2020
A/duck/Bangladesh/43099/2020
A/duck/Bangladesh/43119/2020
A/duck/Bangladesh/44417/2020
A/duck/Bangladesh/44433/2020
A/duck/Bangladesh/44447/2020
A/duck/Bangladesh/43082/2020
A/duck/Bangladesh/43128/2020
A/duck/Bangladesh/44430/2020
A/Common pochard/Bangladesh/42386/2020
A/duck/Bangladesh/44448/2020
A/duck/Bangladesh/43050/2020
A/duck/Bangladesh/43127/2020
A/duck/Bangladesh/44424/2020
A/Ferruginous duck/Bangladesh/42380/2020
A/duck/Bangladesh/44442/2020
A/environment/Bangladesh/42410/2020
A/duck/Bangladesh/43122/2020
A/duck/Bangladesh/44418/2020
A/duck/Bangladesh/44434/2020
A/duck/Bangladesh/44456/2020
A/duck/Bangladesh/43527/2020
A/duck/Bangladesh/44504/2020
A/environment/Bangladesh/42416/2020
A/duck/Bangladesh/43123/2020
A/duck/Bangladesh/44423/2020
A/duck/Bangladesh/44440/2020
A/duck/Bangladesh/44469/2020
A/duck/Bangladesh/44508/2020
A/duck/Bangladesh/44522/2020
A/duck/Bangladesh/43120/2020
A/Whooper swan/Mongolia/24/2020
A/Whooper swan/Mongolia/25/2020
A/Whooper swan/Xinjiang/3/2020
A/Whooper swan/Xinjiang/1/2020
A/Whooper swan/Xinjiang/2/2020
A/Mute swan/Xinjiang/4/2020
A/Mute swan/Xinjiang/5/2020
A/Whooper swan/Xinjiang/6/2020
A/Whooper swan/Xinjiang/7/2020
A/Whooper swan/Xinjiang/8/2020
A/Whooper swan/Xinjiang/9/2020
A/Whooper swan/Xinjiang/10/2020
A/Whooper swan/Xinjiang/11/2020
A/Whooper swan/Xinjiang/12/2020
A/Whooper swan/Xinjiang/13/2020
A/Jiangsu/32888/2018
A/Chongqing/00013/2021
A/Anhui/2021-00011/2020
A/Chicken/Suzhou/j1/2019
A/Env/Guangdong/zhongshan/C172870590/2017-11-28
A/Env/Guangdong/Foshan/C182750024/2018-01-09
A/Guangxi/32797/2018
A/Guangdong/18SF020/2018-09-30
A/Guangxi/31906/2018
A/quail/Vietnam/CVVI-03/2015
A/quail/Vietnam/CVVI-01/2015
A/chicken/Nha Trang/NT3/2017
A/mute swan/Ibaraki/242T/2016
A/mute swan/Ibaraki/242T/2016(2)
A/chicken/Miyagi/1-5T/2017
A/chicken/Kumamoto/1-7T/2016
A/blackheaded gull/Ibaraki/258T/2016
A/Guangxi/55726/2016
A/chicken/Japan/AQ-HE144/2015
A/goose/Hunan/110/2014
A/muscovy/duck/Hunan/148/2014
A/duck/Hunan/12.17 YYFQH0018-Z-P/2014(H5N6)
A/duck/Hunan/HN74/2015
A/duck/Wuhan/WHYF03/2015
A/Environment/Hubei/38005/2014
A/turtledove/Wuhan/WHBJ26/2014
A/duck/Guangdong/03.26 DGCP078-O/2015(H5N6)
A/duck/Guangdong/03.26 DGCPLB007-P/2015(H5N6)
A/muscovy duck/Vietnam/LBM754/2014
A/Ostrich/Guangxi/GX-1/2017(H5N6)
A/Environment/Guangxi/44389/2015
A/Environment/Chongqing/99186/2014
A/Quail/Guangxi/GX-2/2017(H5N6)
A/Env/Guangdong/zhongshan/C182870028/2018-01-15
A/goose/Guangdong/04.22 DGCP117-O/2015(H5N6)
A/duck/Hunan/12.07 YYGK111-P/2013(H5N6)
A/Shoveller/Ningxia/475-11/2015
A/Environment/Chongqing/45373/2015
A/Chicken/Yunnan/YN-10/2015(H5N6)
A/Environment/Chongqing/72096/2014
A/duck/Hunan/02.26 YYFQHY291-P/2014(H5N6)
A/duck/Hunan/12.17 YYGK040/2014(H5N6)
A/duck/Hunan/02.26 YYGK289-P/2014(H5N6)
A/Environment/Guangdong/00299/2014
A/environment/Sichuan/NCLL1/2014
A/duck/Sichuan/04.27 NBXJ717/2014(H5N6)
A/pigeon/Sichuan/04.27 NBXJ743/2014(H5N6)
A/mandarin duck/Korea/H119-3/2017
A/chicken/Kagawa/1C-1/2018
A/duck/Korea/H81/2017
A/pochard duck/England/AVP 18 003254/2018
A/Bird/Netherlands/17017775-035-039/2017
A/Black-headed Gull/Netherlands/29/2017
A/jungle crow/Hyogo/2803A002/2018
A/Jungle crow/Hyogo/2803E024T/2018
A/chicken/Korea/H238/2018
A/duck/Bangladesh/19D853/2017
A/duck/Bangladesh/19D857/2017
A/duck/Bangladesh/19D770/2017
A/goose/Bangladesh/19D764/2017
A/goose/Bangladesh/19D855/2017
A/goose/Bangladesh/19D820/2017
A/duck/Bangladesh/19D851/2017
A/duck/Bangladesh/19D849/2017
A/duck/Bangladesh/19D852/2017
A/Chicken/Jiangxi/45153/2016
A/Chicken/Jiangxi/45093/2016
A/chicken/Jiangxi/04.01 NCJD0010-O/2015(H5N6)
A/chicken/Jiangxi/04.01 NCNP0065-Z-O/2015(H5N6)
A/Environment/Jiangxi/25004/2014
A/environment/Jiangxi/02.10 NCDZT009/2015(H5N6)
0.01

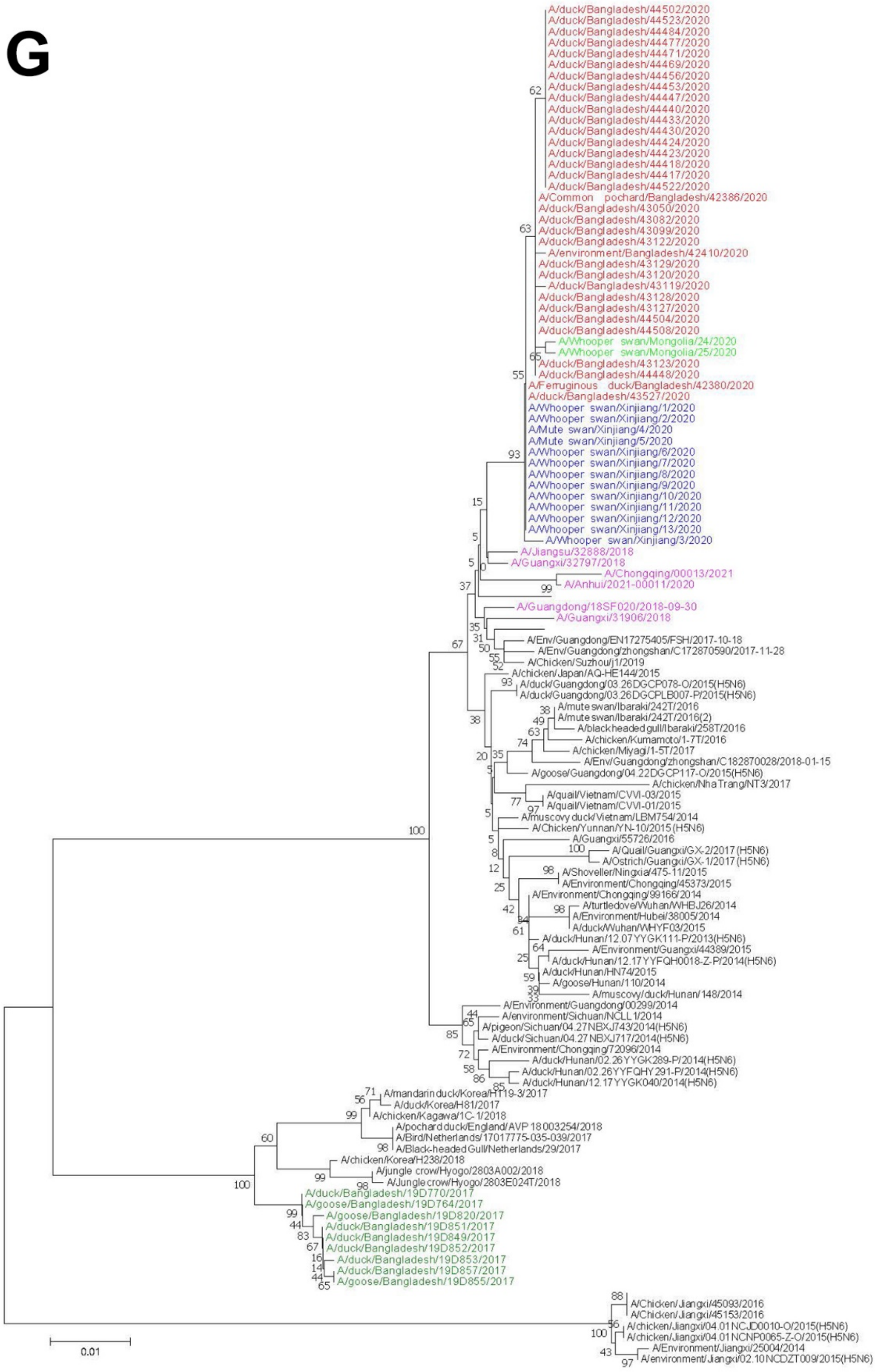

Appendix Figure (following pages). Phylogenetic trees of the A) PB2, B) PB1, C) PA, D) NP, E) NA, F) M and G) NS genes. Phylogenetic analysis was done using the neighbor-joining algorithm with the Kimura 2-parameter model. The reliability of phylogenetic inference at each branch node was estimated by the bootstrap method with 1,000 replications; evolutionary analyses were conducted in MEGA 7 (*5*). HPAI A(H5N6) viruses isolates in this study are marked in red; viruses from Xinjiang, China are marked in blue; viruses from Mongolia are marked in lime; previous H5N6 viruses from Bangladesh are marked in green.
